# β-FeOOH-Loaded Polydopamine-Modified Halloysite Nanotubes as a Catalytic Interlayer for Enhanced Water Dissociation in Bipolar Membranes

**DOI:** 10.3390/membranes16090296

**Published:** 2026-09-10

**Authors:** Haotian Wang, Rui Yang, Jie Wang, Kui Lou, Yunshuang Fan

**Affiliations:** 1 School of Environmental Science and Engineering, Tiangong University, Tianjin 300387, China; 13501196799@163.com (H.W.); yangrui2377@163.com (R.Y.); or7yuki@163.com (J.W.); zhisi11@yeah.net (K.L.); 2 State Key Laboratory of Advanced Separation Membrane Materials, Tiangong University, Tianjin 300387, China

**Keywords:** bipolar membrane, polydopamine, halloysite nanotubes, water dissociation, β-FeOOH

## Abstract

Efficient interfacial water dissociation is essential for reducing the operating voltage of bipolar membranes (BPMs), whereas aggregation and nonuniform distribution of nanoscale catalysts can limit active-site utilization. In this study, polydopamine-modified halloysite nanotubes (PDA@HNTs) were used as a support for β-FeOOH loading to construct a composite catalytic interlayer for BPMs. XRD, FTIR, XPS, SEM, TEM, EDS mapping, contact-angle measurements, electrochemical tests, and bipolar membrane electrodialysis were used to evaluate the interlayer structure and membrane performance. At 50 mA cm^−2^, the β-FeOOH-PDA@HNTs-BPM exhibited a transmembrane voltage of 0.94 V, compared with 2.15 V for the blank BPM, while the interfacial water-dissociation resistance decreased from 3.873 to 0.980 Ω. After 48 h of continuous operation, the voltage increased only from 0.94 to 0.98 V. In electrodialysis, the membrane achieved a current efficiency of 81.4% and an energy consumption of 3.3 kWh kg^−1^ after 180 min. PDA-functionalized HNTs promote the dispersion and interfacial association of β-FeOOH, improve interfacial wettability and catalytic-site accessibility, and thereby enhance water dissociation and acid/base production in BPMs.

## 1. Introduction

A bipolar membrane (BPM) is composed of a cation-exchange layer, an interlayer, and an anion-exchange layer [1,2,3]. Under a reverse electric field, water molecules in the interlayer can dissociate into H^+^ and OH^−^, which migrate through the cation-exchange membrane and anion-exchange membrane into the acid and base compartments, respectively [4,5]. Owing to this feature, bipolar membranes have been widely applied in water electrolysis for hydrogen production [6,7,8,9], CO_2_ reduction [10,11,12,13], electroreductive ammonia synthesis [14], and the recovery of acids and ammonia from nitrogen-containing waste streams [15]. Because water dissociation occurs mainly in the interlayer region between the cation- and anion-exchange layers, the composition, wettability, thickness, and interfacial structure of the interlayer directly affect the water-dissociation performance of BPMs [16,17]. Under the same electric-field conditions, active sites in the intermediate catalytic layer can reduce the activation energy of interfacial water dissociation and accelerate the conversion of water molecules into H^+^ and OH^−^, thereby decreasing water-dissociation resistance and the required transmembrane voltage [18,19].

Based on this principle, researchers have introduced various catalytic components between the anion- and cation-exchange layers of BPMs to reduce the activation energy of water dissociation [20,21]. Reported interlayer catalytic components include hyperbranched polymers containing weakly acidic groups such as carboxyl groups [22], weakly basic materials containing amino or pyridyl groups [4], hydrophilic polymers such as polyethylene glycol and polyvinyl alcohol [23,24], and metal-based catalytic materials such as FeCl_3_ [25], Fe complexes [1], and Fe(OH)_3_ [26]. These catalytic components can promote water polarization and accelerate water dissociation through protonation–deprotonation reactions, Lewis acid–base interactions, or improvement of the interfacial hydration environment. However, for some nanoscale inorganic catalysts, their high surface energy may cause particle aggregation during membrane fabrication and operation, resulting in a decrease in effective active surface area and nonuniform interfacial distribution; some catalytic components may also migrate or leach from the interlayer [25,27,28].

To further address these issues, hollow or porous catalytic materials and support-assisted immobilization strategies have attracted increasing attention. For example, hollow SnO_2_ microspheres can increase the effective reaction interface through their open structure [3], while amino-functionalized MIL-101 can provide a porous framework [29]. A bipolar membrane cationic layer constructed using polydopamine-coated kaolin nanotubes loaded with phosphotungstic acid has been reported to improve the mechanical stability, hydrophilicity, and ion-transport performance of the membrane layer [30]. Previous studies have shown that in situ construction of α-FeOOH in the BPM interlayer can significantly reduce the water-dissociation energy barrier and improve operational stability [31]. β-FeOOH is also an iron oxyhydroxide material, and its surface contains abundant hydroxyl groups and diverse Fe coordination environments, which may provide potential active sites for water adsorption and interfacial proton transfer [32]. The catalytic performance of FeOOH depends not only on its intrinsic activity but also strongly on its interfacial dispersion and immobilization state. Therefore, selecting a support with an appropriate structure and tunable surface chemistry can facilitate FeOOH loading while suppressing its aggregation. Halloysite nanotubes (HNTs) possess a one-dimensional hollow tubular structure [33,34], which provides a potential structural basis for the spatial dispersion of nanoscale catalytic components. Polydopamine (PDA) can form a uniform coating rich in polar functional groups on the HNT surface [30], thereby regulating its surface chemistry and wettability [35,36]. Therefore, loading β-FeOOH onto PDA-functionalized HNTs is expected to simultaneously improve catalytic-phase dispersion, the interfacial hydration environment, and active-site accessibility, thereby enhancing catalytic performance.

Based on the above analysis, β-FeOOH was loaded onto PDA-functionalized HNTs to construct a β-FeOOH-PDA@HNTs composite catalytic interlayer, and BPMs were fabricated by a casting method. Four groups of BPMs, namely those without an interlayer and those containing HNTs, PDA@HNTs, or β-FeOOH-PDA@HNTs, were prepared to progressively investigate the effects of HNT introduction, PDA surface functionalization, and β-FeOOH loading on membrane performance. By analyzing the catalytic-layer composition, catalytic-phase dispersion, and interfacial interaction characteristics, together with indicators including the onset of water dissociation, interfacial impedance, operational stability, current efficiency, and energy consumption, the effects of catalytic-layer structure on BPM water dissociation and electrodialytic acid/base production were evaluated. This study aims to establish the relationship among composite-interlayer composition, catalytic-phase dispersion, and interfacial water-dissociation performance, and to provide a reference for the design of iron-based catalytic interlayers for BPMs with low water-dissociation resistance.

## 2. Materials and Methods

### 2.1. Materials and Reagents

Halloysite nanotubes were purchased from Guangdong Jina New Materials Technology Co., Ltd. (Foshan, China); dopamine hydrochloride (DA) was obtained from Shanghai Zhengfan Technology Co., Ltd. (Shanghai, China); ferric chloride hexahydrate was obtained from Beijing Innochem Science & Technology Co., Ltd. (Beijing, China); sodium chloride, hydrochloric acid, anhydrous sodium carbonate, sodium hydroxide, and N,N-dimethylformamide (DMF) were obtained from Tianjin Fengchuan Chemical Reagent Technology Co., Ltd. (Tianjin, China); anhydrous ethanol, methanol, and anhydrous sodium sulfate were obtained from Tianjin Kermel Chemical Reagent Co., Ltd. (Tianjin, China) Unless otherwise stated, all chemicals were of analytical grade and were used as received without further purification. Deionized water was used in all experiments.

The cation-exchange membrane used in the experiments was sulfonated poly(phenylene oxide) purchased from Shandong Tianwei Membrane Technology Co., Ltd. (Weifang, China), while the anion-exchange membrane was self-prepared quaternized poly(phenylene oxide). In the electrochemical performance tests, the anion-exchange membrane (FUJI-TYPE 12 AEM) and cation-exchange membrane (FUJI-TYPE 12 CEM) used as electrodialysis separators together with the BPM were supplied by Fuji, Tokyo, Japan, and the proton-exchange membrane (Nafion 117) was supplied by DuPont, Wilmington, DE, USA. Their performance parameters are listed in Table 1.

### 2.2. Preparation of Bipolar Membranes

#### 2.2.1. Preparation of the Anion-Exchange Membrane Casting Solution

Quaternized poly(phenylene oxide) (QPPO) was prepared according to the literature [37], with a quaternization degree of approximately 35%. A total of 1 g of the prepared QPPO was added to a mixed solvent of methanol and DMF (QPPO 5 wt%, DMF 15 wt%, and methanol 80 wt%). After magnetic stirring at room temperature for 12 h, the resulting solution was used as the anion-exchange membrane casting solution.

#### 2.2.2. Preparation of Interlayer Materials

(1) PDA@HNTs: A 0.01 mol L^−1^ Tris-HCl buffer solution was prepared according to reported methods [38,39]. Dopamine (0.1 g) was added to 100 mL of the Tris-HCl buffer solution and ultrasonicated for 30 min. Subsequently, 0.5 g of HNTs was added and the suspension was magnetically stirred at 25 °C for 24 h. The resulting solid was collected by centrifugation and washed three times with deionized water to remove unreacted dopamine and free PDA species. Finally, the product was dried at 60 °C for 12 h, yielding 0.52 g of PDA@HNTs powder.
(2) β-FeOOH-PDA@HNTs: FeCl_3_·6H_2_O (5.56 g) was dissolved in 100 mL of deionized water. After complete dissolution, 10 mL of ethanol and 1.2 g of NaOH were added sequentially. The mixture was magnetically stirred at 90 °C for 30 min. After stirring, the precipitate was collected by centrifugation and washed twice each with deionized water and ethanol. The precipitate was then dried in an oven at 60 °C for 24 h, yielding 1.6 g of yellow β-FeOOH powder. Subsequently, 0.10 g of β-FeOOH was dispersed in 20 mL of deionized water to form a suspension, and 0.10 g of PDA@HNTs powder was added to the β-FeOOH suspension. After magnetic stirring for 4 h, the product was collected by centrifugation, washed three times with deionized water and three times with ethanol, and dried at 60 °C for 24 h, yielding 0.18 g of β-FeOOH-PDA@HNTs powder.

#### 2.2.3. Fabrication of Bipolar Membranes

**Figure 1:**
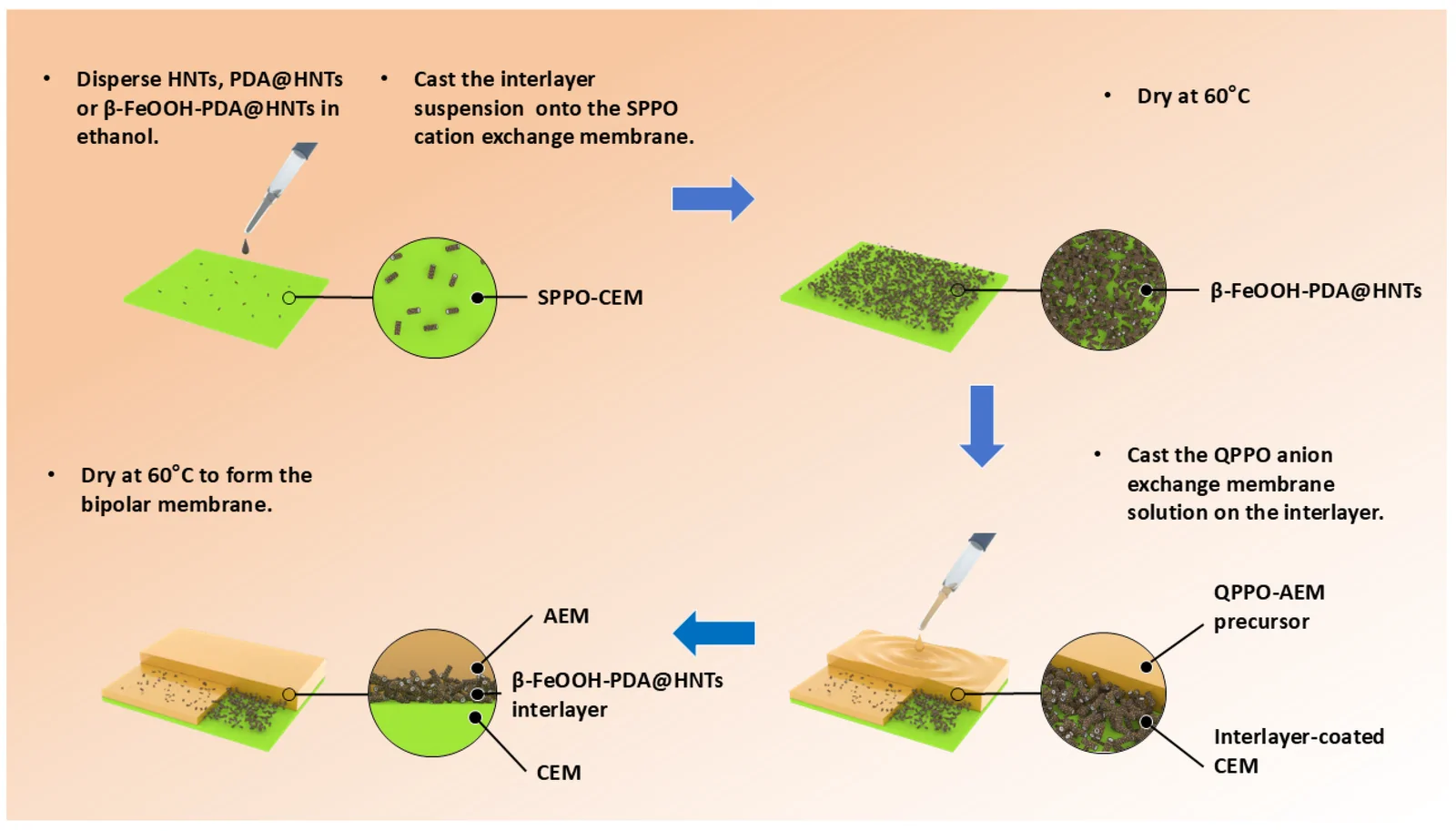
Schematic fabrication procedure of β-FeOOH-PDA@HNTs-BPM.

HNTs, PDA@HNTs, and β-FeOOH-PDA@HNTs powders (0.1 g each) were separately dispersed in 10 mL of ethanol to prepare the corresponding interlayer dispersions. The cation-exchange membrane was cut into 3 cm × 3 cm pieces and used as the base membrane. Each base membrane was laid flat on a glass plate, and 1 mL of the corresponding interlayer dispersion was measured and cast onto the cation-exchange membrane using a dropper, followed by drying at 60 °C for 1 h. Subsequently, 1 mL of the anion-exchange membrane casting solution was cast onto the interlayer and dried at 60 °C for 12 h to obtain the BPM. For comparison, a BPM without an interlayer was prepared by directly casting the anion-exchange membrane casting solution onto the cation-exchange membrane and was used as the blank control. The overall fabrication procedure is illustrated in Figure 1.

### 2.3. Characterization and Performance Testing of Bipolar Membranes

#### 2.3.1. Characterization

The morphologies of HNTs, PDA@HNTs, and β-FeOOH-PDA@HNTs were observed using scanning electron microscopy (SEM, TESCAN MIRA LMS, Brno, Czech Republic). Cross-sections of the BPM samples were obtained by brittle fracture in liquid nitrogen and sputter-coated with gold before testing. Energy-dispersive X-ray spectroscopy (EDS) was used to analyze the elemental distribution across the membrane cross-sections. Transmission electron microscopy (TEM, JEOL JEM-F200, Tokyo, Japan) was further used to observe the microstructure and loading state of β-FeOOH-PDA@HNTs. The crystal phase of β-FeOOH was analyzed using an X-ray diffractometer (XRD, D8 DISCOVER, Bruker, Karlsruhe, Germany) with Cu Kα radiation over a 2θ range of 10–80° at a scanning rate of 20° min^−1^. Functional groups were analyzed using a Fourier-transform infrared spectrometer (Nicolet iS50, Thermo Fisher Scientific, Waltham, MA, USA) in ATR mode over a wavenumber range of 4000–400 cm^−1^. The surface elemental composition and chemical states of the samples were analyzed by X-ray photoelectron spectroscopy (XPS, NEXSA, Thermo Fisher Scientific, USA) using Al Kα as the excitation source, and the binding energies were calibrated using the C 1s peak at 284.8 eV. Peak fitting was performed using Avantage software, version 6.9.0. Static water contact angles of different interlayer surfaces were measured using an automatic contact-angle analyzer (DSA30S, KRÜSS, Hamburg, Germany). The water-droplet volume was set to 1 μL, and each sample was measured five times, and the results are reported as mean ± standard deviation (n = 5).

#### 2.3.2. Performance Tests

(1) I–V Curve Measurements

**Figure 2:**
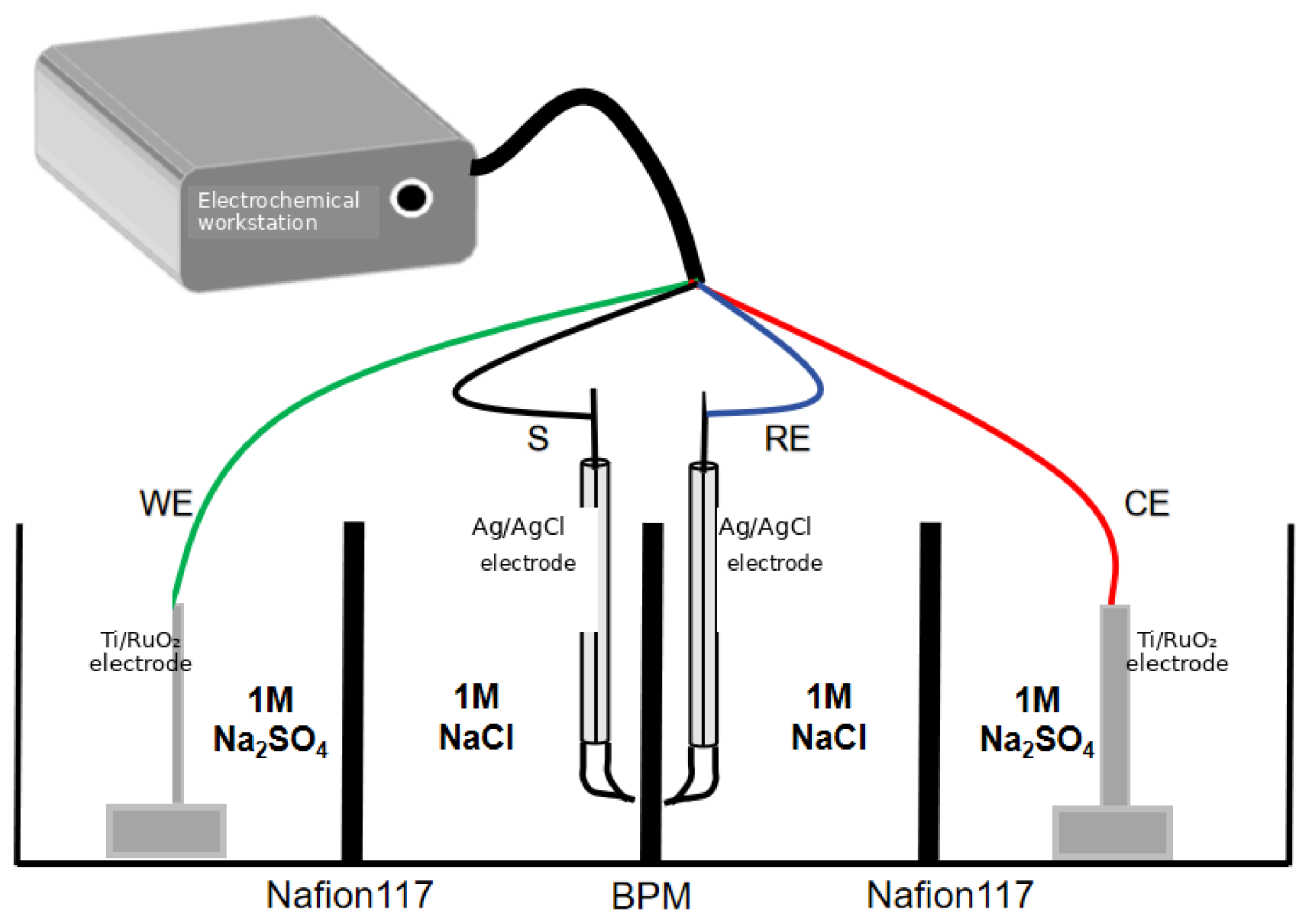
Schematic diagram of the electrochemical testing setup for bipolar membranes.

The I–V curves of the BPMs were measured using an electrochemical workstation (PGSTAT302N, Herisau, Switzerland). The fabricated BPM was fixed in the middle of the compartment with an effective area of 9 cm^2^, and measurements were performed over a current-density range of 0–200 mA cm^−2^. The acid and base compartments adjacent to the BPM contained 1 mol L^−1^ NaCl solution, while the anode and cathode compartments contained 1 mol L^−1^ Na_2_SO_4_ as the electrolyte. Two commercial Nafion 117 membranes were used to separate the electrode compartments from the acid and base compartments. The electrochemical workstation was connected to the compartment, a pair of Ti/RuO_2_ electrodes supplied the current, and two Ag/AgCl electrodes collected the electrochemical signals of the test sample. A schematic diagram of the electrochemical testing setup is shown in Figure 2. For the I–V measurements, one independently prepared membrane specimen of each BPM type was tested (n = 1); therefore, these electrochemical data are presented as representative measurements without statistical error bars.

(2) Electrochemical Impedance Spectroscopy Measurements

The electrochemical impedance spectroscopy measurements of the BPMs were performed using the same setup and solution system as those used for the I–V measurements. The IMP-A.C. Impedance mode was selected. The open-circuit voltage of the BPM was first measured, and the scanning frequency range was set to 10^5^–10^−2^ Hz. The obtained data were imported into ZView software and fitted using an appropriate equivalent circuit. Under reverse bias, the catalytic interfacial layer of the BPM is considered to be almost completely depleted of mobile ions, leading to the formation of a depletion region. The thickness of the interfacial depletion region (λ) can be obtained from the fitted equivalent capacitance, and the time required for ions to migrate out of the interfacial layer (τ_tr_) can be calculated from the frequency in the Bode plot. The calculation equations are as follows:

(1) $$\lambda =\frac{\varepsilon_{o}\varepsilon_{r}A_{ef}}{C}$$

(2) $$\tau_{tr}=\frac{1}{2\pi f_{max}}$$

where ε_0_ is the vacuum permittivity, 8.854 × 10^−12^ F m^−1^; ε_r_ is the dielectric constant of the sample, taken as 80 for pure water; A_ef_ is the effective contact area; C is the equivalent capacitance obtained from ZView; f_max_ is the frequency corresponding to the maximum value on the *y*-axis of the Bode plot. For the EIS measurements, one independently prepared membrane specimen of each BPM type was tested (n = 1); therefore, these electrochemical data are presented as representative measurements without statistical error bars.

(3) Constant-Current Operational Stability Test of Bipolar Membranes

Chronopotentiometry was used to monitor the transmembrane voltage of the BPMs under constant-current conditions and to investigate its variation with time. The setup was the same as that used for the I–V measurements. The Autolab program was set to Chronopotentiometry mode, the test current density was 50 mA cm^−2^, the voltage recording interval was 180 s, and the total test duration was 48 h. The 48 h constant-current stability test was performed using one independently prepared membrane specimen for each BPM formulation (n = 1), and the resulting voltage–time curves are presented as representative continuous-operation measurements.

(4) Bipolar Membrane Electrodialysis Test

**Figure 3:**
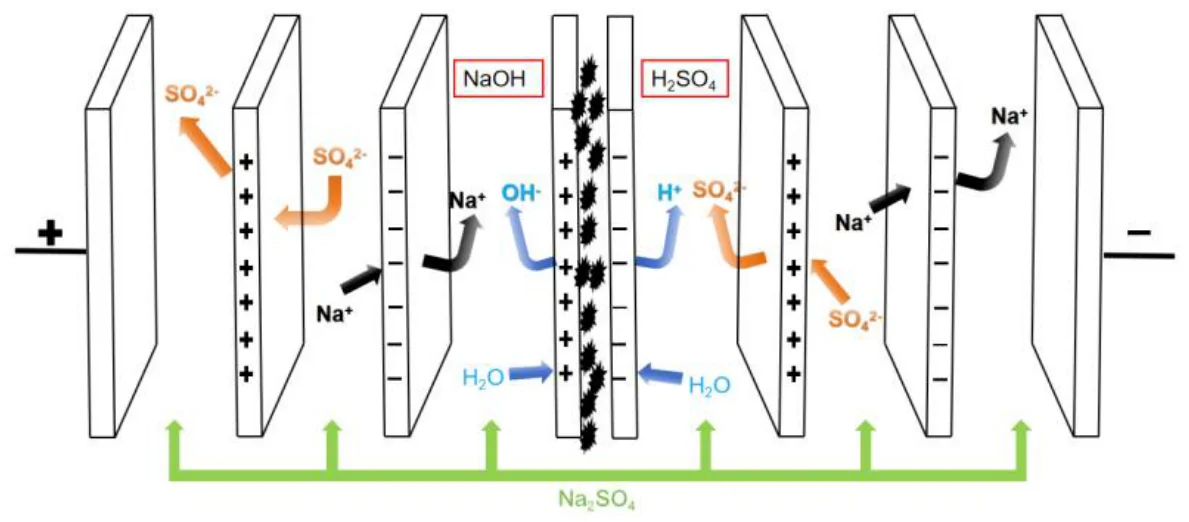
Schematic diagram of the acid/base production setup.

Bipolar membrane electrodialysis was performed using a self-built six-compartment apparatus with an A-C-BP-A-C (AMX-CMX-BPM-AMX-CMX) membrane-stack configuration. The effective contact area of the BPM was 9 cm^2^. From left to right, the compartments were arranged as electrode compartment, salt compartment, base compartment, acid compartment, salt compartment, and electrode compartment. The configuration of the bipolar membrane electrodialysis system is illustrated in Figure 3. Each compartment contained 200 mL of 1 mol L^−1^ Na_2_SO_4_ as the electrolyte solution. Throughout the experiment, a stable constant current was supplied at a current density of 50 mA cm^−2^ (0.45 A). The OH^−^ and H^+^ generated by water dissociation in the BPM passed through the anion- and cation-exchange membranes, respectively, into the adjacent compartments, where they combined with Na^+^ and SO_4_^2−^ to form NaOH and H_2_SO_4_. Every 30 min, 2 mL of solution was withdrawn from the acid and base compartments to determine the acid/base concentration, and the membrane-stack voltage was recorded at the same time. The experiment lasted for 3 h. Acid and base concentrations were determined by titration; 0.05 mol L^−1^ HCl and Na_2_CO_3_ solutions were used to titrate the base and acid, respectively, with phenolphthalein as the indicator. The current efficiency (η) of acid/base production and the electrodialysis energy consumption (E) were calculated to evaluate BPM performance using the following equations:

(3) $$\eta =\frac{(C_{t}V_{t}-C_{0}V_{0})F}{NIt}\times 100\%$$

(4) $$E=\int_{0}^{t}\frac{U_{t}Idt}{(C_{t}V_{t}-C_{0}V_{0})M}$$

where C_t_ and C_0_ are the H^+^ concentrations in the acid compartment at time t and at the initial time, respectively (mol L^−1^); V_t_ and V_0_ are the corresponding solution volumes at time t and initially (L); F is the Faraday constant (96,485 C mol^−1^); N is the number of repeating units (N = 1); I is the applied current (A); t is the operating time (s); Ut is the total membrane-stack voltage at time t (V); and M is the molar mass of H_2_SO_4_ (g mol^−1^). Bipolar membrane electrodialysis experiments were independently repeated using three separately prepared membrane specimens for each BPM type (n = 3), and the results are reported as mean ± standard deviation.

## 3. Results and Discussion

### 3.1. Characterization of Bipolar Membrane Interlayer Materials

**Figure 4:**
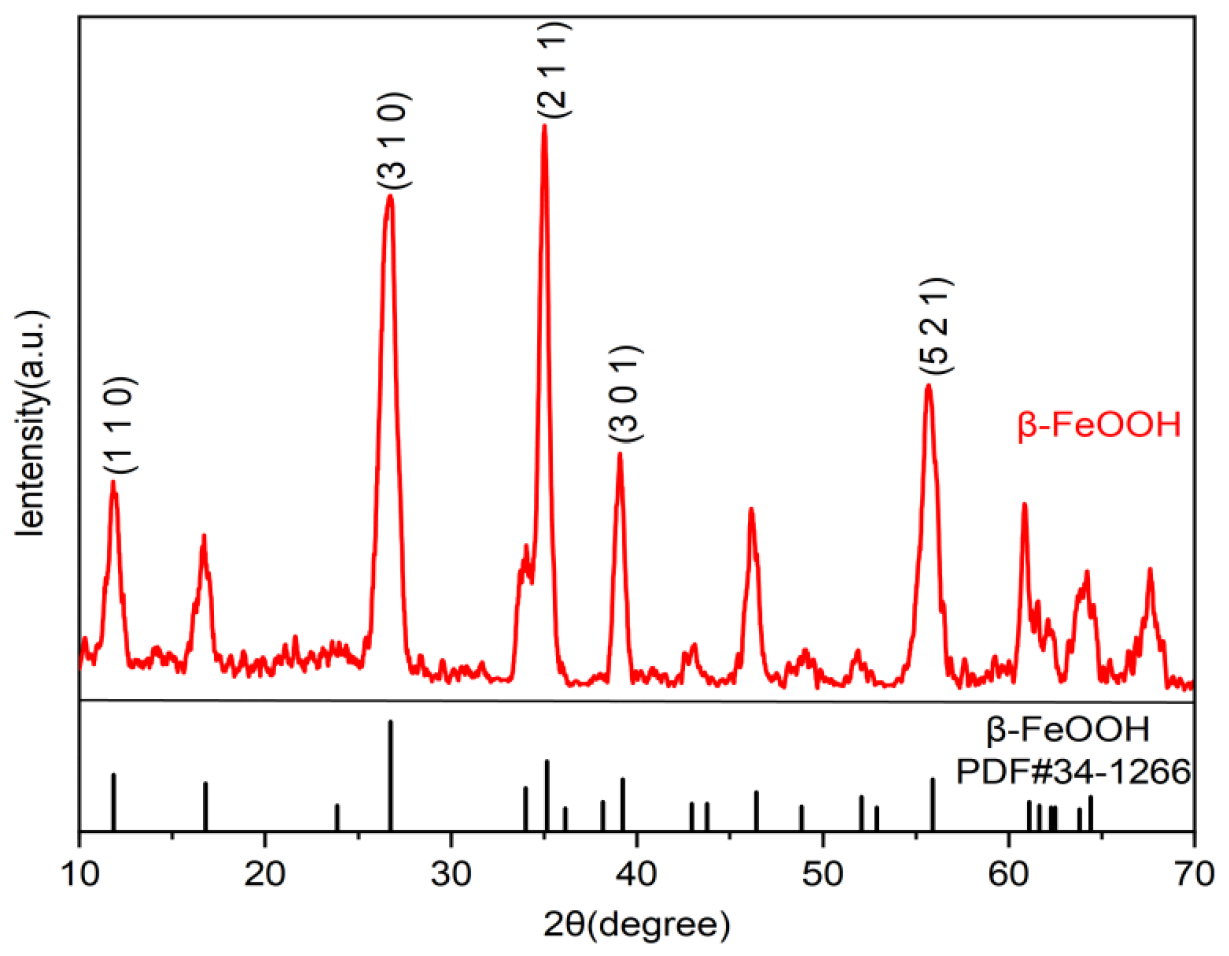
X-ray diffraction pattern of β-FeOOH.

To verify whether β-FeOOH was synthesized by the hydrothermal method, X-ray diffraction (XRD) was used for phase analysis. As shown in Figure 4, pronounced diffraction peaks were observed at 2θ values of 11.8°, 26.7°, 35.2°, 39.2°, and 55.9°. Their positions are highly consistent with the diffraction data of the (1 1 0), (3 1 0), (2 1 1), (3 0 1), and (5 2 1) crystal planes in the standard β-FeOOH card [40] (PDF#34-1266). The sharp peak shapes and relatively small full widths at half maximum indicate a high degree of crystallinity. Full-pattern fitting using Jade software did not reveal obvious characteristic peaks of α-FeOOH (goethite) or γ-FeOOH (lepidocrocite), such as those at 2θ = 21.2° and 33.2°, indicating that the obtained product was predominantly β-phase. These results demonstrate that the product was mainly β-FeOOH, providing a phase basis for its subsequent loading onto HNTs.

**Figure 5:**
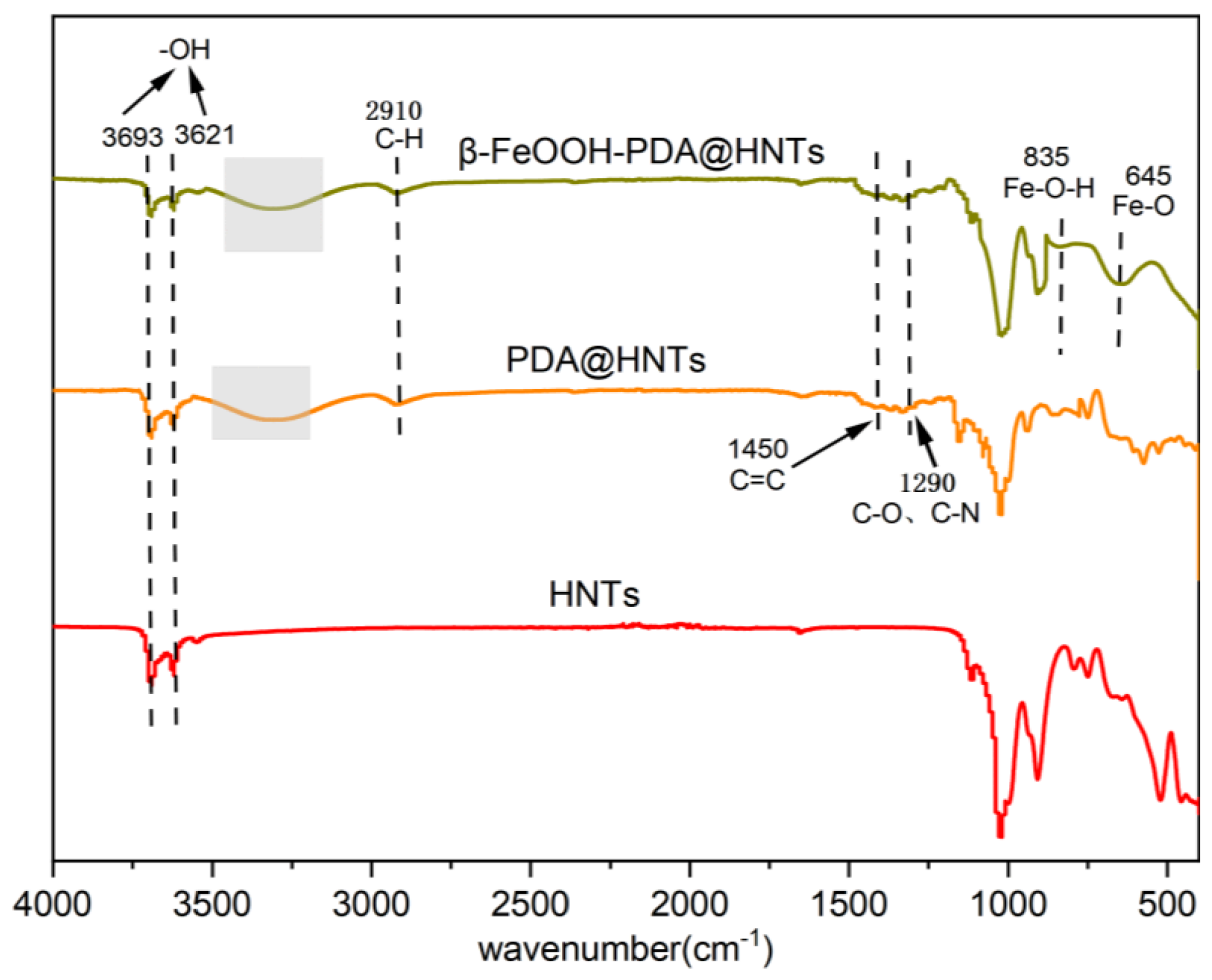
FTIR spectra of HNTs, PDA@HNTs, and β-FeOOH-PDA@HNTs.

**Figure 6:**
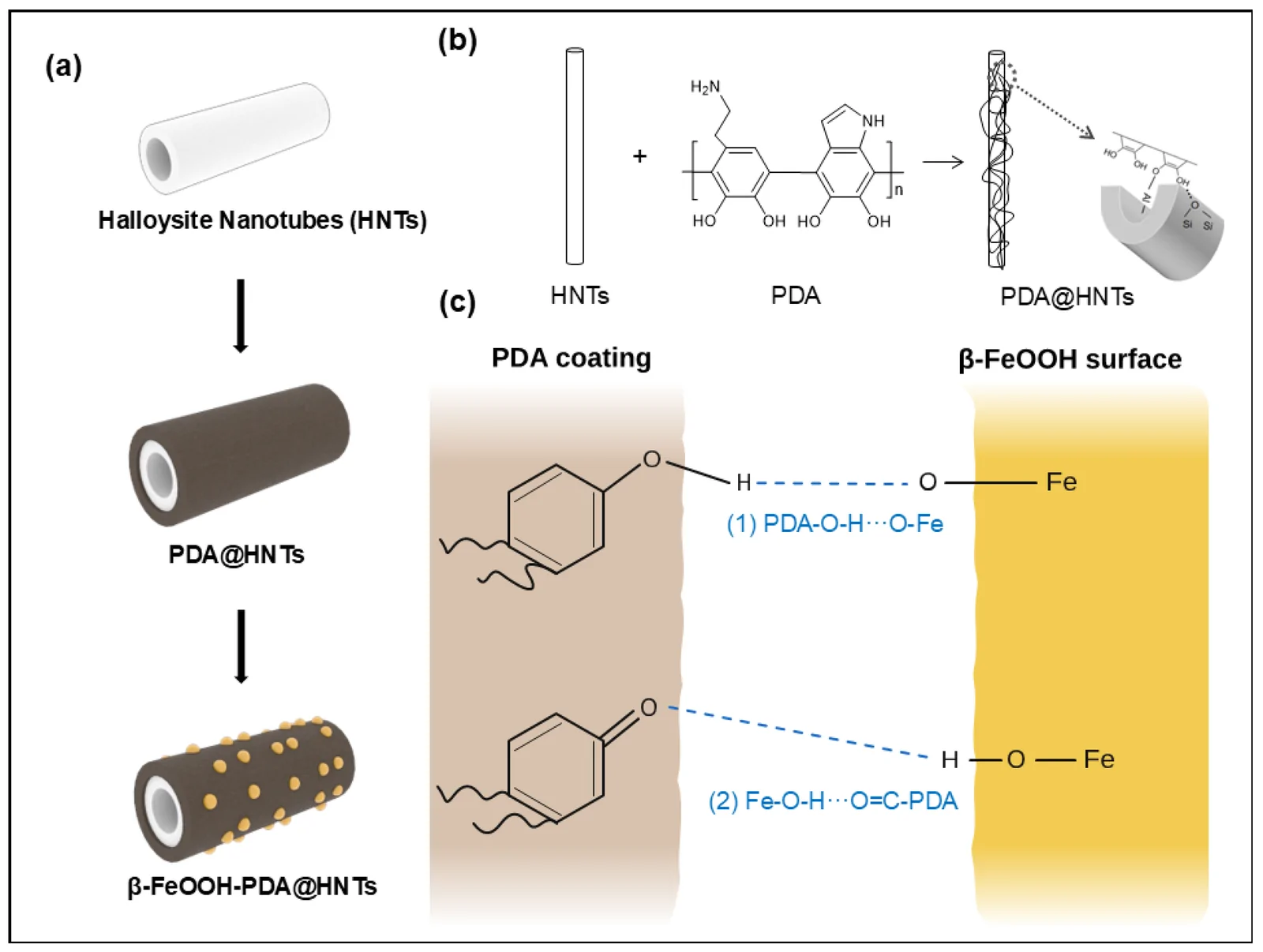
Schematic illustration of the construction process and interfacial interactions of the β-FeOOH-PDA@HNTs composite: (**a**) stepwise construction of β-FeOOH-PDA@HNTs; (**b**) schematic reaction process of PDA on the HNT surface; and (**c**) proposed interfacial interactions between PDA and β-FeOOH.

Figure 5 shows the FTIR spectra of HNTs, PDA@HNTs, and β-FeOOH-PDA@HNTs. In the HNT spectrum, the peaks at 3693, 3621, and 908 cm^−1^ correspond to the stretching vibration of external surface –OH groups, the stretching vibration of internal Al–OH groups, and the bending vibration of Al–OH groups, respectively. The peaks at 1015 and 454 cm^−1^ are assigned to the stretching and bending vibrations of Si–O (Si–O–Si and O–Si–O), respectively. After PDA modification, a broad band formed at 3300 cm^−1^, while new peaks appeared at 2910 and 1290 cm^−1^, indicating changes in the organic functional-group environment on the HNT surface and supporting the introduction of PDA. The construction process and interfacial interactions of the PDA@HNTs composite are shown in Figure 6a,b. Under weakly alkaline conditions, dopamine undergoes oxidative self-polymerization to form PDA. Polar functional groups such as catechol –OH groups interact with Si–O–Si and Al–OH sites on the HNT surface through hydrogen bonding and surface adsorption, forming a PDA coating on the HNT surface. After further loading with β-FeOOH, β-FeOOH-PDA@HNTs showed an Fe–O lattice symmetric vibration at 645 cm^−1^; the strong absorption in this region can serve as a characteristic fingerprint of β-FeOOH. The Fe–O–H band at 835 cm^−1^ indicates the introduction of an iron oxyhydroxide phase into the PDA@HNTs composite [41]. The FTIR results are consistent with the XRD results, confirming the successful construction of the β-FeOOH-PDA@HNTs composite.

**Figure 7:**
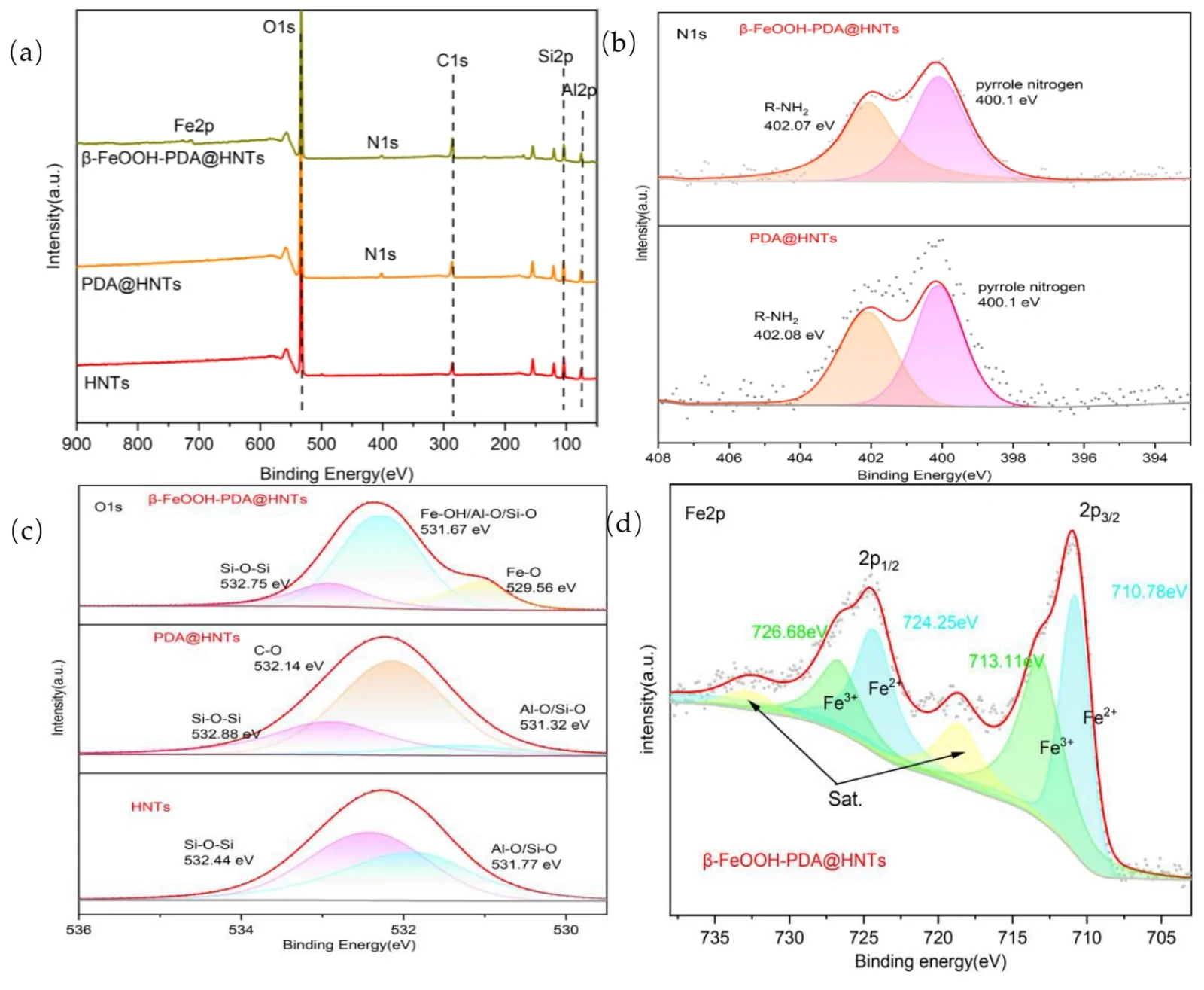
(**a**) Survey spectra of HNTs, PDA@HNTs, and β-FeOOH-PDA@HNTs; (**b**) N 1s spectra of PDA@HNTs and β-FeOOH-PDA@HNTs; (**c**) O 1s spectra of HNTs, PDA@HNTs, and β-FeOOH-PDA@HNTs; and (**d**) Fe 2p spectrum of β-FeOOH-PDA@HNTs.

Figure 7a shows the XPS survey spectra of HNTs, PDA@HNTs, and β-FeOOH-PDA@HNTs. As shown in Figure 7a, HNTs mainly exhibit Al, Si, and O signals (the C signal originates from the conductive adhesive). After PDA modification, a characteristic N signal appeared in the PDA@HNTs spectrum and the C 1s signal increased, indicating that a nitrogen-containing organic component was introduced onto the HNT surface. After further loading with β-FeOOH, an Fe 2p signal appeared in the survey spectrum of β-FeOOH-PDA@HNTs, indicating successful introduction of the iron-based component into the composite. Figure 7b shows the high-resolution N 1s spectra of PDA@HNTs and β-FeOOH-PDA@HNTs. According to the current peak-fitting results, the N 1s signal of PDA@HNTs can be deconvoluted into two nitrogen-containing components at approximately 400.1 and 402.08 eV. After β-FeOOH loading, the binding energies and relative peak shapes of the N 1s components did not change markedly, indicating limited changes in the local chemical environment of the nitrogen-containing groups in PDA. Figure 7c shows the high-resolution O 1s spectra of HNTs, PDA@HNTs, and β-FeOOH-PDA@HNTs. The O 1s spectrum of HNTs is mainly composed of oxygen-containing structures such as Si–O–Si and Al–O/Al–OH. After PDA modification, the O 1s peak shape and relative areas of the components changed, indicating that the oxygen-containing chemical environment on the HNT surface was affected by the PDA coating. After β-FeOOH loading, a new low-binding-energy component appeared at approximately 529.56 eV, which can be assigned to lattice Fe–O in the iron oxyhydroxide phase. The enhanced component near 531.67 eV may arise from the combined contributions of surface Fe–OH groups in β-FeOOH and the pre-existing hydroxyl/oxygen-containing species. As illustrated in Figure 6c, the abundant phenolic hydroxyl groups and other oxygen-containing functional groups on the PDA surface may interact with β-FeOOH through hydrogen bonding, surface adsorption, and other interfacial interactions, thereby facilitating the loading and dispersion of β-FeOOH on PDA@HNTs. Therefore, these results support the successful incorporation of β-FeOOH into PDA@HNTs and suggest that the oxygen-containing functional groups on PDA may participate in the interfacial interactions between β-FeOOH and the support. Characteristic Fe signals were observed in the β-FeOOH-PDA@HNTs spectrum, and the high-resolution spectrum is shown in Figure 7d. Owing to spin–orbit coupling, the high-resolution Fe 2p XPS spectrum exhibits typical split peaks. Considering that β-FeOOH is an Fe(III) oxyhydroxide, the Fe species in the present composite are predominantly assigned to Fe(III). The Fe 2p envelope exhibits characteristic spin–orbit splitting together with a complex multiplet structure. The components located at approximately 710.78 and 713.11 eV are associated with the Fe 2p3/2 region, whereas those at approximately 724.25 and 726.68 eV are located in the Fe 2p1/2 region. The accompanying satellite features further support the presence of Fe(III)-containing iron oxyhydroxide. Similar Fe 2p binding energies have been reported for β-FeOOH, with Fe 2p3/2 and Fe 2p1/2 signals typically appearing at approximately 711–712 and 724–726 eV, respectively [42]. Together with the characteristic β-FeOOH diffraction peaks in XRD and the Fe–O and Fe–OH vibrational bands in FTIR, the XPS results further support the successful incorporation of β-FeOOH into PDA@HNTs.

**Figure 8:**
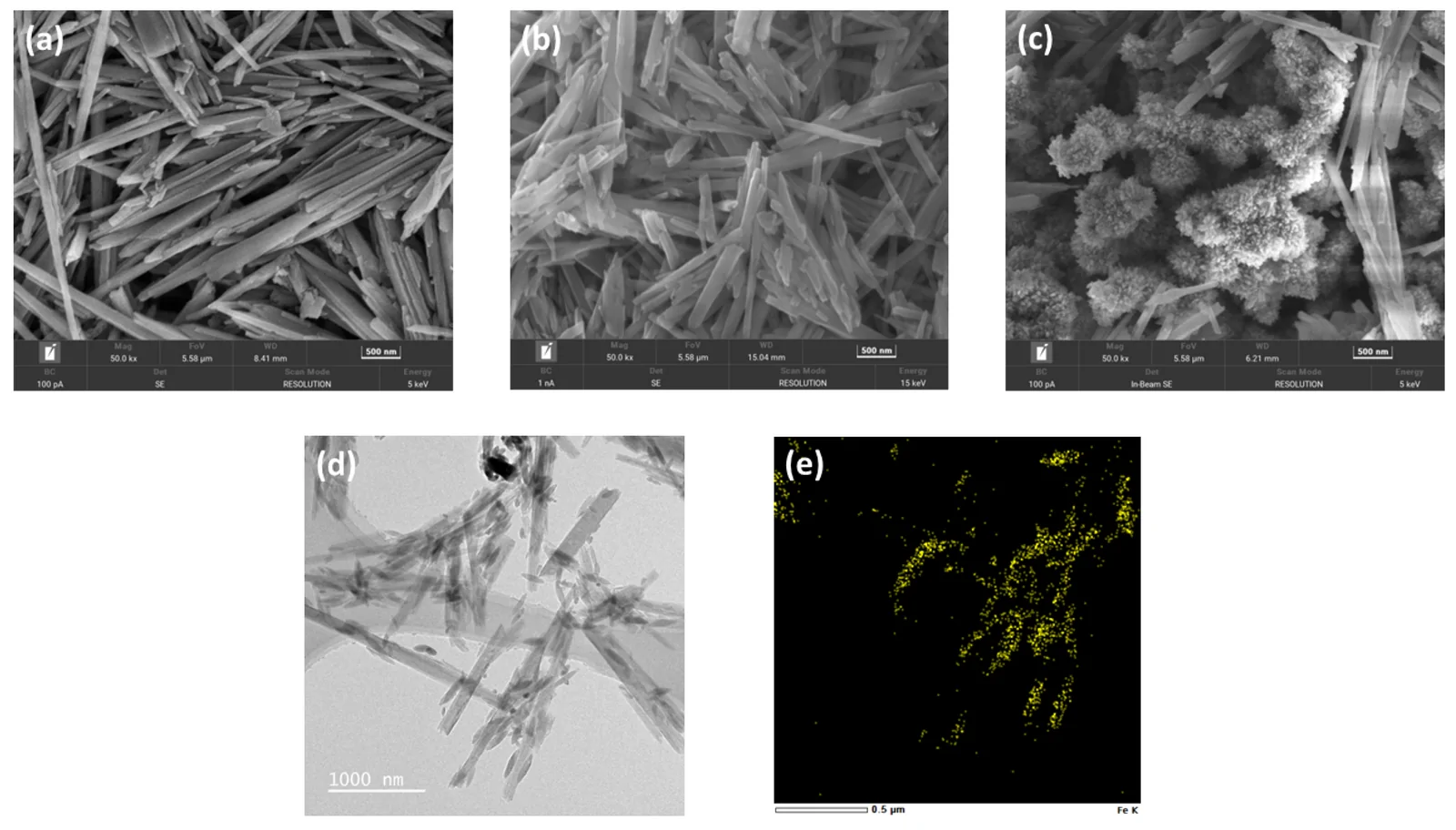
SEM images of (**a**) HNTs, (**b**) PDA@HNTs, and (**c**) β-FeOOH-PDA@HNTs; (**d**) TEM image of β-FeOOH-PDA@HNTs; and (**e**) EDS mapping of Fe.

To analyze the effects of PDA modification and β-FeOOH loading on the morphology of HNTs, the three materials were characterized by SEM and TEM. As shown in Figure 8a, pristine HNTs exhibit a hollow tubular structure with open ends, relatively smooth unloaded tube walls, lengths of 1–2 μm, and outer diameters of approximately 70–100 nm. The material is mainly composed of O, Al, and Si (C originates from testing contamination), and the Al/Si content ratio is close to 1:1, which is highly consistent with the stoichiometric relationship of HNTs, [Al_2_Si_2_O_5_(OH)_4_·nH_2_O]. After PDA modification, the basic tubular morphology of the HNTs was retained, but the tube-wall surface became rougher (Figure 8b), indicating that the surface coating altered the morphological characteristics of the HNTs. After further loading with β-FeOOH, needle-like or particulate species could be observed around the HNTs (Figure 8c). TEM and Fe elemental-distribution results showed that β-FeOOH crystals were distributed around the HNT walls, with no obvious free crystals observed within the examined field of view (Figure 8d,e). These results indicate that PDA-functionalized HNTs can serve as a dispersing support for β-FeOOH, helping to suppress direct aggregation among β-FeOOH crystals and improve the interfacial dispersion of catalytic active sites.

**Figure 9:**
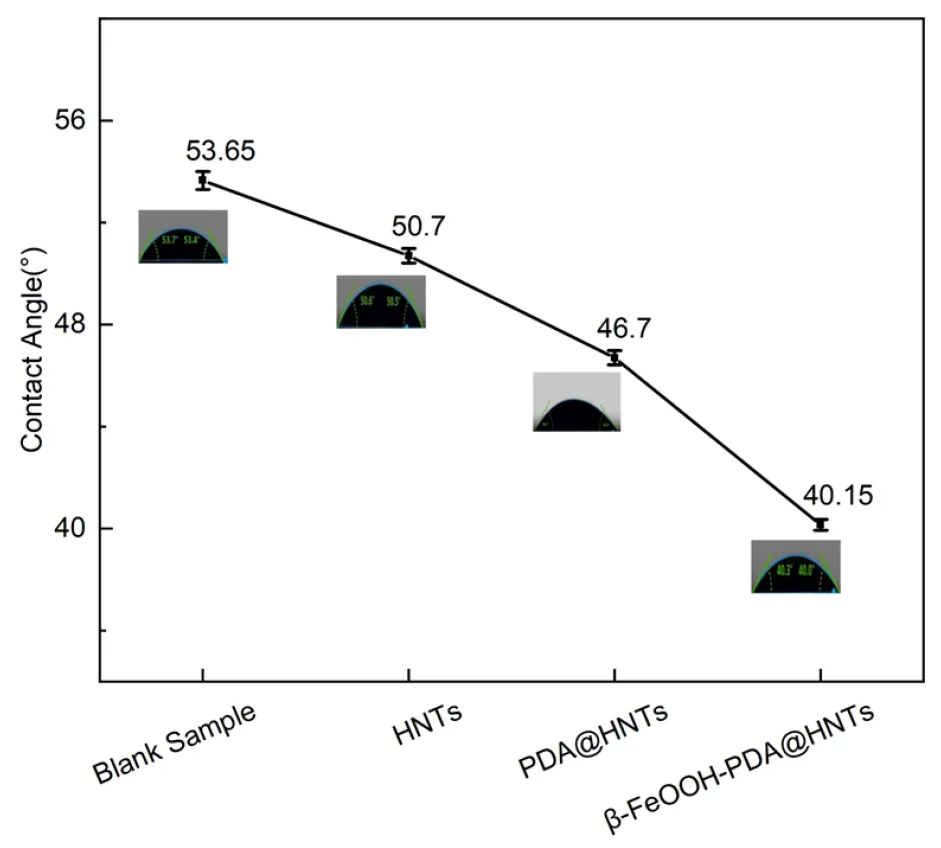
Water contact angles of the interfacial surfaces without an interlayer and with HNTs, PDA@HNTs, and β-FeOOH-PDA@HNTs interlayers. Data are presented as mean ± standard deviation from five measurements (n = 5).

Figure 9 shows the static water contact angles of different interlayer surfaces. The contact angle of Blank-BPM was 53.65°, which decreased to 50.7° after the introduction of HNTs and further decreased to 46.7° after PDA modification. The β-FeOOH-PDA@HNTs interlayer exhibited the lowest contact angle, 40.15°. The progressive decrease in contact angle indicates that PDA surface functionalization and β-FeOOH introduction improved the wettability of the interlayer. This may be related to the abundant polar functional groups on the surfaces of PDA and β-FeOOH. Improved wettability facilitates the approach of water molecules to the catalytic interface and provides a hydrated environment for subsequent interfacial water dissociation and ion migration [43].

### 3.2. Cross-Sectional Analysis of Bipolar Membranes

**Figure 10:**
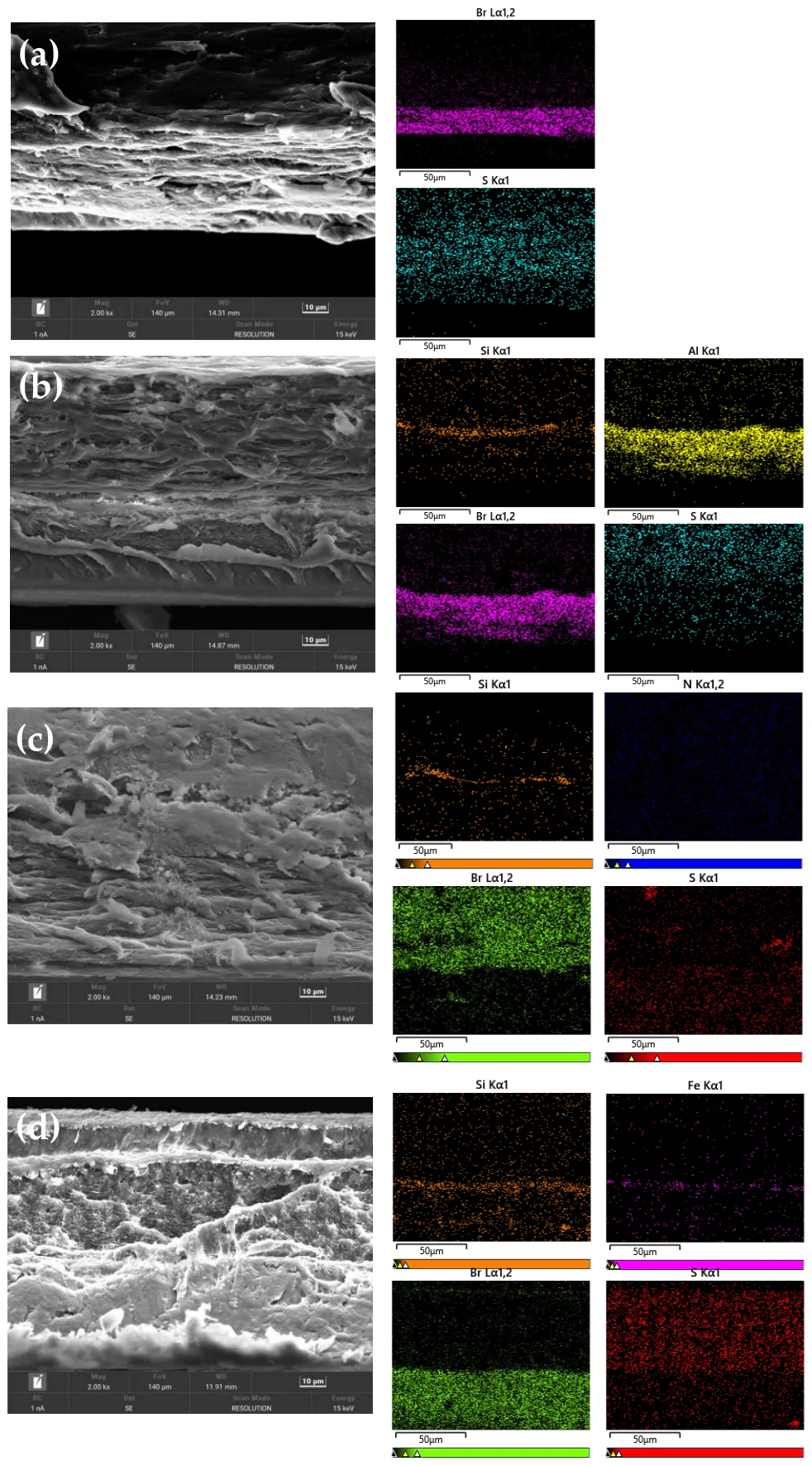
Cross-sectional SEM images and corresponding EDS elemental maps of different bipolar membranes: (**a**) Blank-BPM; (**b**) HNTs-BPM; (**c**) PDA@HNTs-BPM; and (**d**) β-FeOOH-PDA@HNTs-BPM. The triangles (▲) indicate the minimum and maximum intensity thresholds (color scale limits) set by the EDS software.

To clarify the distribution of the composite interlayer at the BPM interface, the cross-sectional morphologies and elemental distributions of different BPMs were analyzed by SEM-EDS. The corresponding cross-sectional SEM images and elemental maps are presented in Figure 10. For the blank bipolar membrane without an interlayer (Blank-BPM), cross-sectional EDS showed that Br was mainly concentrated in the lower layer. This is because –Br was not completely quaternized during BPPO synthesis, and residual –Br on the QPPO backbone was detected, indicating that this layer was the anion-exchange membrane. The characteristic element in the upper layer was S, originating from –SO3− groups on the SPPO backbone, indicating that this layer was the cation-exchange membrane. After the introduction of HNTs, HNTs-BPM exhibited the typical three-layer structure of a bipolar membrane, with interlayer particles embedded between the anion- and cation-exchange layers. EDS mapping showed that the interfacial region mainly contained Si and Al. Because the Al and Br peaks are close in energy, their characteristic peaks overlap and are difficult to distinguish by EDS; nevertheless, the presence of Si also confirms the successful introduction of halloysite nanotubes. The interfacial region of PDA@HNTs-BPM likewise showed clear Si enrichment accompanied by a relatively weak N signal, indicating that PDA-functionalized HNTs were mainly distributed between the anion- and cation-exchange layers. The relatively weak N signal may be related to the low PDA content in the overall membrane cross-section and interference from the matrix background. For β-FeOOH-PDA@HNTs-BPM, Si and Fe enrichment was most pronounced near the bipolar junction, although weak Fe-related signals were also detected in the adjacent membrane regions, indicating that the β-FeOOH-PDA@HNTs composite was successfully constructed in the BPM interlayer. Compared with Blank-BPM, introduction of the composite interlayer made the interfacial morphology rougher, while the overall structure remained a relatively continuous three-layer architecture without obvious large-scale interfacial defects. The increased interfacial roughness may increase the effective contact area between the interlayer and the two adjacent membrane layers, while the distribution of the composite in the interfacial region provides a structural basis for water molecules to access catalytic sites and for the migration of water-dissociation products [44,45].

In summary, the cross-sectional SEM and EDS results confirm the successful introduction of HNTs, PDA@HNTs, and β-FeOOH-PDA@HNTs composites at the interface between the anion- and cation-exchange layers and show that BPMs containing an interlayer possess a continuous three-layer structure in the initial state.

### 3.3. Electrochemical Performance Analysis of Bipolar Membranes

**Figure 11:**
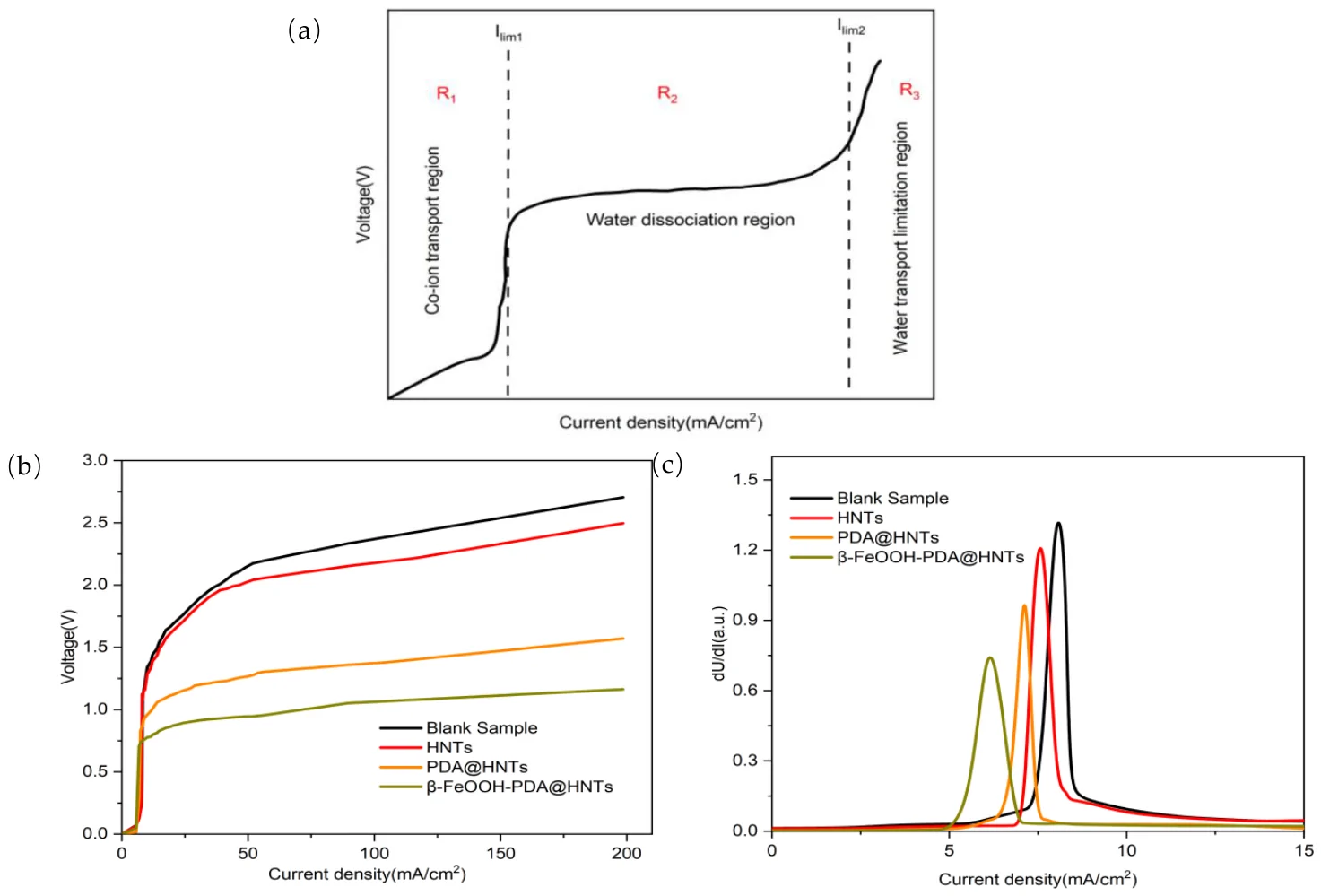
(**a**) Characteristic I–V curve of a BPM under neutral conditions; (**b**) I–V curves of Blank-BPM, HNTs-BPM, PDA@HNTs-BPM, and β-FeOOH-PDA@HNTs-BPM; and (**c**) dU/dI curves. The I–V data are representative measurements from one independently prepared membrane specimen for each BPM type (n = 1).

The transmembrane voltage is an important parameter for evaluating BPM performance. Figure 11 presents the I–V curves of the four BPMs and the dU/dI plots obtained by differentiating the I–V curves. The I–V curves show that introduction of the catalyst reduced the transmembrane voltage of the BPMs. At a current density of 50 mA cm^−2^, the transmembrane voltage of the blank BPM was 2.15 V and decreased to 2.04 V after the introduction of HNTs, possibly because of improved interlayer wettability. Xue demonstrated that the interfacial water content is an important factor affecting BPM water-dissociation performance [46]; therefore, introduction of HNTs promoted water dissociation and reduced the transmembrane voltage. After PDA modification, the transmembrane voltage decreased further, possibly because polar functional groups on the PDA surface improved the interfacial hydration environment while participating in protonation–deprotonation reactions [47], thereby reducing the water-dissociation resistance and transmembrane voltage. β-FeOOH-PDA@HNTs-BPM exhibited the lowest transmembrane voltage, 0.94 V. For comparison, Shehzad et al. reported that the commercial Neosepta BP1 exhibited a transmembrane voltage of approximately 1.2 V at 50 mA cm^−2^ under comparable reverse-bias water-dissociation conditions, as estimated from the reported I–V curve [31]. Although differences in electrolyte concentration, effective membrane area, and cell configuration should be considered, this comparison indicates that the β-FeOOH-PDA@HNTs-BPM developed in this work exhibits competitive water-dissociation performance. Combined with the FTIR, XPS, and SEM/TEM results, β-FeOOH contains hydroxyl sites with multiple coordination environments; their proton affinity and hydrogen-bonding environment may affect water adsorption and interfacial proton-transfer processes [32]. Meanwhile, HNTs favor improved dispersion of β-FeOOH, thereby increasing the number of accessible catalytic sites and reducing the voltage required for interfacial water dissociation. The limiting current density (I_lim_) is another important parameter for characterizing BPM performance. Under a reverse electric field, the I–V curve of a BPM can be divided into three regions [48,49], as shown in Figure 11a. R_1_ is the co-ion migration region, which arises from the incomplete ion selectivity of the anion- and cation-exchange layers of the BPM; R_2_ is the water-dissociation region, formed by the water-dissociation reaction in the BPM; and R_3_ is the water-depletion region, which occurs when the water-consumption rate caused by dissociation in the interfacial layer greatly exceeds the rate at which water diffuses into the interface, resulting in drying of the BPM interfacial layer. A characteristic current point at the boundary between R_1_ and R_2_ is defined as the first limiting current density, I_lim1_, corresponding to the onset current for water dissociation. A lower I_lim1_ indicates that water dissociation occurs more readily, whereas I_lim2_ is associated with water deficiency in the interfacial layer. Figure 11c was obtained by differentiating the I–V curves, and the current density corresponding to the maximum value on the *y*-axis is I_lim1_. As summarized in Table 2, the I_lim1_ values of Blank-BPM, HNTs-BPM, PDA@HNTs-BPM, and β-FeOOH-PDA@HNTs-BPM were 8.13, 7.98, 7.13, and 5.80 mA cm^−2^, respectively. With the successive introduction of HNTs, PDA, and β-FeOOH, I_lim1_ progressively decreased. β-FeOOH-PDA@HNTs-BPM exhibited the lowest I_lim1_, indicating that the catalytic layer reduced the onset current for water dissociation and made the water-dissociation process easier. The absence of I_lim2_ indicates good hydrophilicity of the BPM and suggests that no interfacial water depletion occurred during water dissociation.

**Figure 12:**
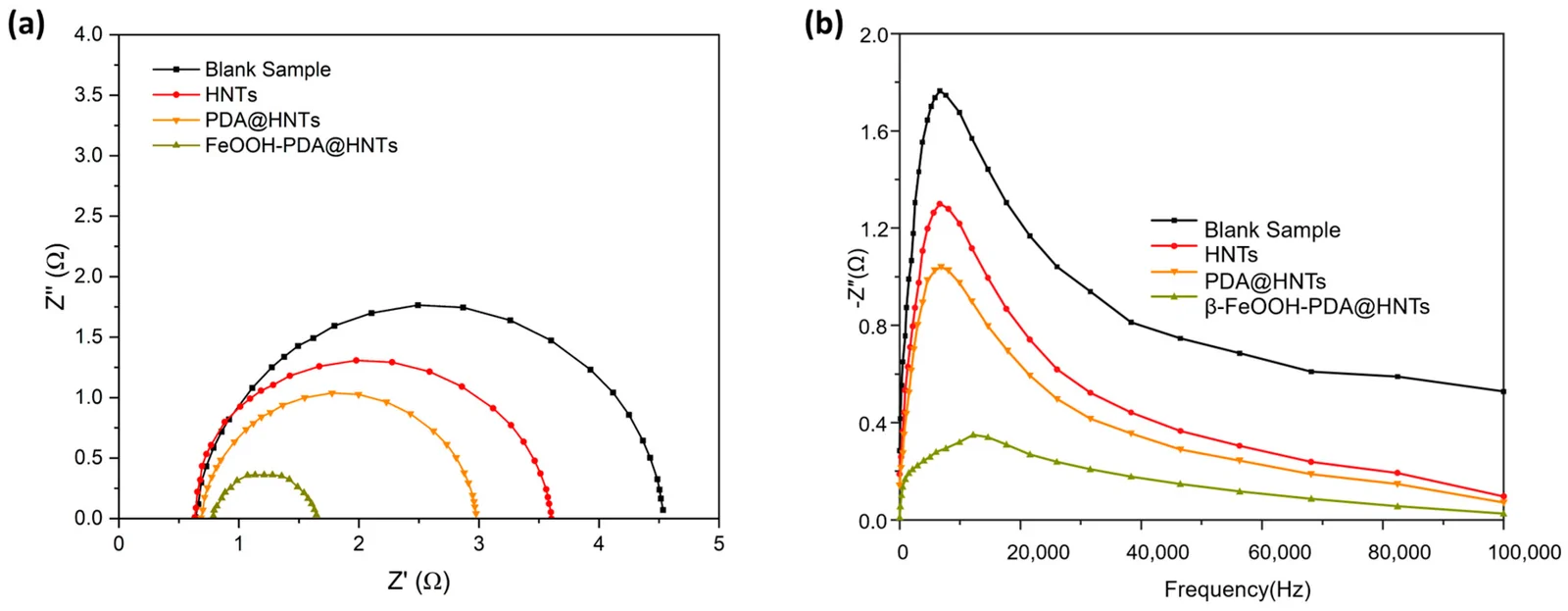
Electrochemical impedance characteristics of Blank-BPM, HNTs-BPM, PDA@HNTs-BPM, and β-FeOOH-PDA@HNTs-BPM: (**a**) Nyquist plots and fitted equivalent circuit; and (**b**) Bode plots. The EIS data are representative measurements from one independently prepared membrane specimen for each BPM type (n = 1).

To further distinguish the ohmic resistance of the BPM from the resistance associated with interfacial water dissociation and to analyze the effect of the composite catalytic interlayer on interfacial ion-transport kinetics, electrochemical impedance spectroscopy (EIS) measurements were performed on the different BPMs. The Nyquist and Bode plots of the different BPMs are shown in Figure 12. The equivalent circuit used for fitting the EIS data consisted of two components connected in series [49]: an ohmic resistance element R_1_, followed by a parallel circuit composed of a water-dissociation resistance element R_w_ and a constant-phase element (CPE). R_1_ represents the total ohmic resistance of the anion- and cation-exchange layers before water dissociation occurs and corresponds to the value from the origin to the first intersection of the test curve with the *x*-axis. R_w_ represents the water-dissociation resistance of the BPM interfacial layer during water dissociation and corresponds to the distance between the two intersections of the semicircle with the *x*-axis. A smaller semicircle diameter indicates a lower resistance associated with interfacial water dissociation. The CPE is a constant-phase element used to describe non-ideal capacitive behavior caused by factors such as interfacial roughness and compositional heterogeneity. As shown in Table 3, the R_1_ values of Blank-BPM, HNTs-BPM, PDA@HNTs-BPM, and β-FeOOH-PDA@HNTs-BPM were 0.627, 0.650, 0.680, and 0.780 Ω, respectively. R_1_ increased slightly with the progressive introduction of the composite interlayer, indicating that interlayer particles and newly introduced interfaces may contribute additional ohmic resistance. In contrast to the trend in R_1_, the R_w_ values were 3.873, 2.950, 2.290, and 0.980 Ω, respectively. With the successive introduction of HNTs, PDA, and β-FeOOH, the interfacial water-dissociation resistance continuously decreased, and the R_w_ of β-FeOOH-PDA@HNTs-BPM was only approximately 25% of that of the blank membrane. Although the composite interlayer caused a slight increase in R_1_, the decrease in R_w_ was much greater than the increase in ohmic resistance, indicating that promotion of interfacial water-dissociation kinetics by the composite interlayer was dominant. Consequently, β-FeOOH-PDA@HNTs-BPM still exhibited the lowest transmembrane voltage in the I–V measurements. The BPM containing the β-FeOOH-PDA@HNTs catalyst exhibited the smallest semicircle radius and the lowest resistance to water dissociation, consistent with the I–V analysis. This indicates that hydrophilic –OH groups on the β-FeOOH surface improved water permeability, promoted water splitting, and reduced the water-dissociation resistance. The calculated thickness of the depletion region (λ) and the time required for ions to migrate out of the interfacial layer (τtr) are listed in Table 3. Compared with the blank BPM, β-FeOOH-PDA@HNTs showed markedly reduced depletion-region thickness and ion migration time, reaching 9.82 nm and 13.1 μs, respectively. This is attributed to the interlayer catalyst, which increases the generation rates and fluxes of H^+^ and OH^−^, compensates for the imbalance of fixed charges in the anion/cation membrane layers, and causes the depletion region to contract, thereby reducing its thickness and shortening the time required for ions to migrate out of the interfacial layer [50]. The reduced depletion-region thickness and shortened migration time indicate that the composite catalytic interlayer enhanced the generation and migration rates of interfacial water-dissociation products and alleviated interfacial charge depletion. Overall, the I–V and EIS results show that, although β-FeOOH-PDA@HNTs-BPM has a slightly higher ohmic resistance, its interfacial water-dissociation resistance, first limiting current density, depletion-region thickness, and characteristic migration time are all significantly reduced. These results indicate that the composite interlayer promotes BPM water dissociation mainly by improving the interfacial hydration environment, increasing catalytic-site accessibility, and accelerating the interfacial ionic response.

**Figure 13:**
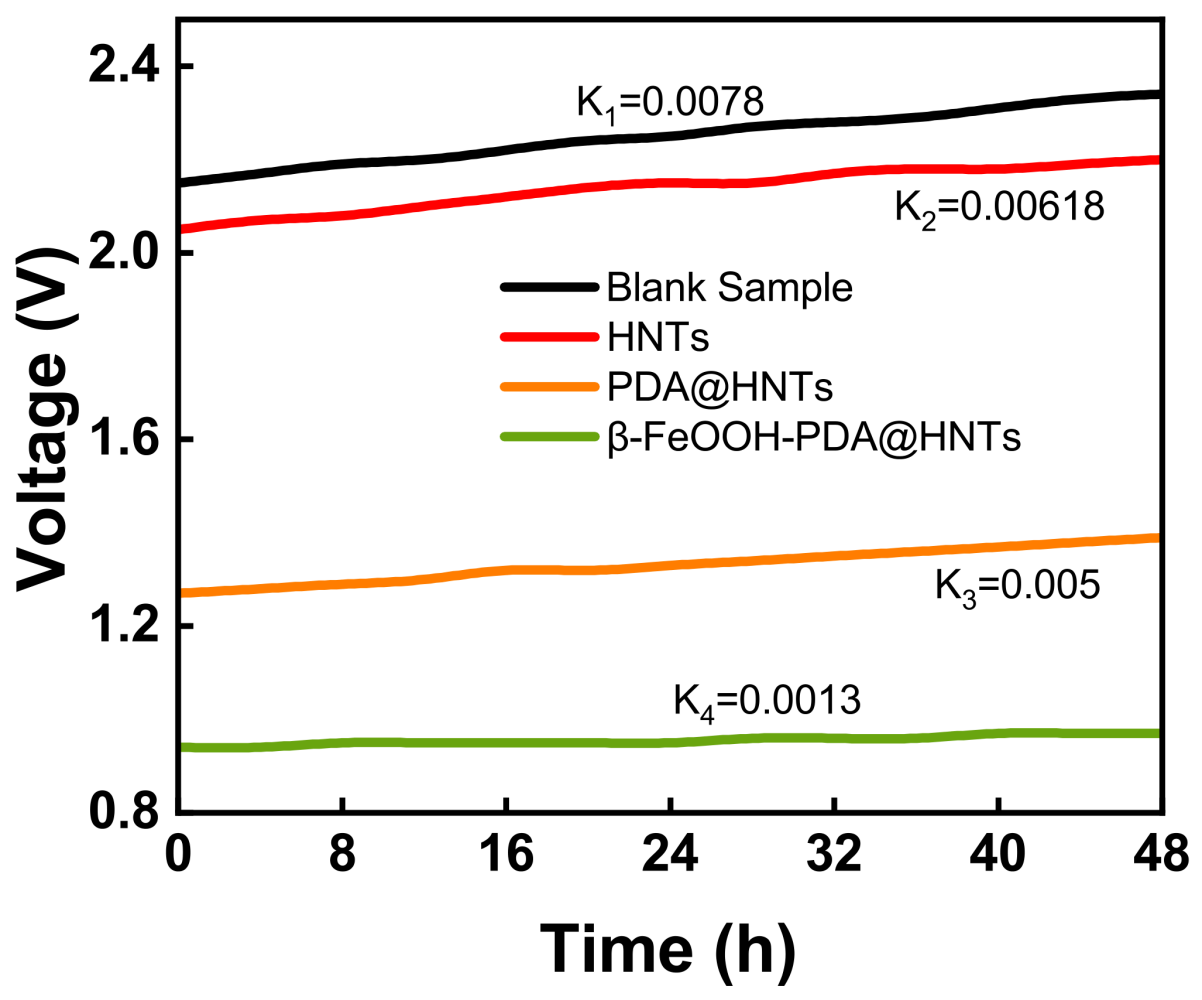
Transmembrane-voltage variation of Blank-BPM, HNTs-BPM, PDA@HNTs-BPM, and β-FeOOH-PDA@HNTs-BPM during continuous operation for 48 h at 50 mA cm^−2^. The voltage–time curves are representative continuous-operation measurements from one independently prepared membrane specimen for each BPM type (n = 1).

To evaluate the performance retention of BPMs with different catalytic interlayers during continuous water dissociation, the transmembrane voltage was monitored continuously for 48 h at a constant current density of 50 mA cm^−2^, as shown in Figure 13. During 48 h of constant-current operation, the transmembrane voltages of all BPMs changed to different extents. The voltage of Blank-BPM increased from 2.15 to 2.34 V. With the introduction of HNTs and PDA functionalization, the overall transmembrane voltage decreased and the magnitude of voltage variation during operation was also reduced. After loading β-FeOOH, the initial transmembrane voltage of β-FeOOH-PDA@HNTs-BPM was 0.94 V and increased to only 0.98 V after 48 h, corresponding to a small change of 0.04 V, while remaining at a low level throughout the test period. This indicates that the composite catalytic interlayer maintained relatively stable water-dissociation performance under constant-current operation for 48 h. Combined with the structural characterization results discussed above, the improved dispersion of β-FeOOH on PDA-functionalized HNTs and the possible interfacial interactions between β-FeOOH and the support may contribute to maintaining an effective catalytic interface.

### 3.4. Bipolar Membrane Electrodialysis Performance

**Figure 14:**
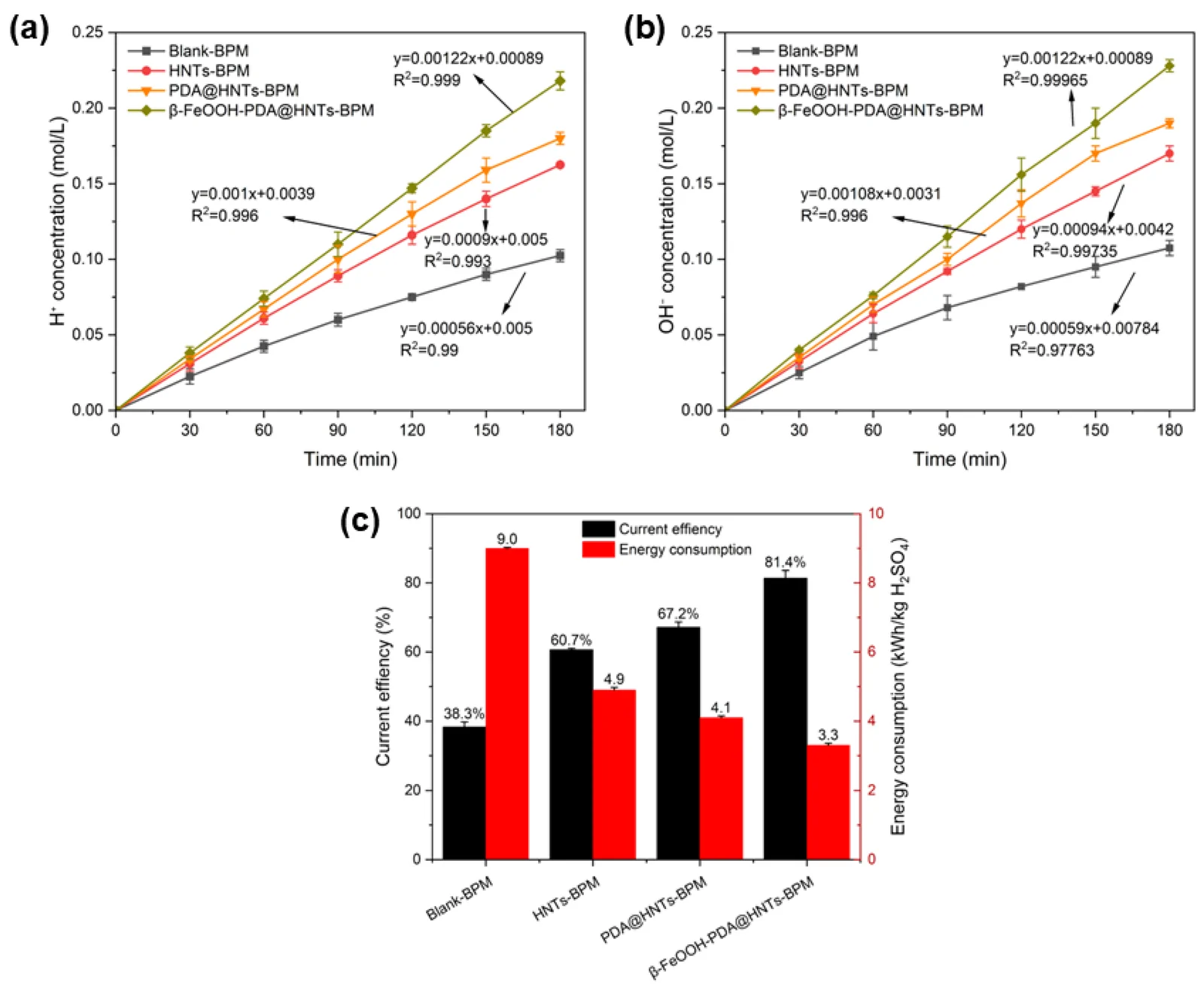
Electrodialysis performance of Blank-BPM, HNTs-BPM, PDA@HNTs-BPM, and β-FeOOH-PDA@HNTs-BPM at 50 mA cm^−2^: (**a**) H^+^ concentration in the acid compartment as a function of time; (**b**) OH^−^ concentration in the base compartment as a function of time; and (**c**) current efficiency and energy consumption per unit acid production after 180 min of operation. Data are presented as mean ± standard deviation from three independent membrane specimens (n = 3).

To evaluate the effect of different catalytic interlayers on the practical acid/base production capacity of the BPMs, bipolar membrane electrodialysis experiments were conducted for 180 min at a constant current density of 50 mA cm^−2^. The changes in acid and base concentrations with operating time were analyzed, and the current efficiency and energy consumption per unit acid production were calculated, as shown in Figure 14. As shown in Figure 14a,b, the H^+^ concentration in the acid compartment and the OH^−^ concentration in the base compartment continuously increased for all BPMs during 180 min of operation, indicating that all of the BPMs could continuously generate H^+^ and OH^−^ under a reverse electric field. Compared with Blank-BPM, introduction of HNTs, PDA@HNTs, and β-FeOOH-PDA@HNTs interlayers progressively increased the acid and base concentrations, with β-FeOOH-PDA@HNTs-BPM exhibiting the highest concentrations. For all samples, the OH^−^ concentration was slightly higher than the corresponding H+ concentration. This difference may result from the combined effects of differences in permselectivity between the anion- and cation-exchange membranes and H+ leakage. After 180 min, the mean H^+^ and OH^−^ concentrations of Blank-BPM were 0.1025 and 0.1075 mol L^−1^, respectively; those of PDA@HNTs-BPM reached 0.180 and 0.190 mol L^−1^, respectively; and those of β-FeOOH-PDA@HNTs-BPM further increased to 0.218 and 0.228 mol L^−1^, respectively. The introduction of HNTs may facilitate the approach of water molecules to the interface by improving interfacial wettability. Amino and phenolic hydroxyl groups on the PDA surface act as water-dissociation active sites, further improve the interfacial hydration environment, and may participate in protonation–deprotonation processes. After β-FeOOH loading, the Fe–OH-rich iron oxyhydroxide interface and the dispersion of β-FeOOH around the HNT walls favor improved catalytic-site accessibility, thereby significantly enhancing acid/base production.

As shown in Figure 14c, the current efficiency of β-FeOOH-PDA@HNTs-BPM reached 81.4 ± 0.2%, higher than the 38.3 ± 0.2% of Blank-BPM. Its energy consumption per unit acid production was 3.3 kWh kg^−1^, substantially lower than the 9.0 kWh kg^−1^ of Blank-BPM. The improved current efficiency indicates that, under the same applied current, a greater fraction of the input charge was effectively used for the generation and migration of H^+^ and OH^−^. The reduced energy consumption is mainly associated with the lower transmembrane voltage and interfacial water-dissociation resistance of the composite membrane after catalyst introduction. In summary, introduction of the water-dissociation interlayer not only increased the acid/base production concentration of the BPMs but also improved current efficiency and reduced energy consumption.

β-FeOOH-PDA@HNTs-BPM simultaneously exhibited the highest acid/base production concentrations and current efficiency and the lowest energy consumption per unit product. These results are consistent with its lowest transmembrane voltage in the I–V measurements and lowest interfacial water-dissociation resistance in the EIS measurements, indicating that improvement of interfacial water-dissociation kinetics by the composite catalytic interlayer can be translated into enhanced acid/base production performance during bipolar membrane electrodialysis. Taken together, the I–V, EIS, and BPMED results show that the lower interfacial water-dissociation resistance of β-FeOOH-PDA@HNTs-BPM leads to higher acid/base production capacity and current utilization efficiency, thereby reducing the energy consumption per unit product.

## 4. Conclusions

In this study, a β-FeOOH composite catalytic interlayer for bipolar membranes was constructed using PDA-functionalized HNTs as the support. Characterization confirmed that β-FeOOH was successfully introduced and dispersed around the HNT walls, while the interfacial wettability of the composite interlayer was improved. At 50 mA cm^−2^, the transmembrane voltage and interfacial water-dissociation resistance of β-FeOOH-PDA@HNTs-BPM decreased from 2.15 V and 3.873 Ω for the blank membrane to 0.94 V and 0.980 Ω, respectively. The performance improvement of the BPM mainly resulted from the extensive introduction and successful dispersion of β-FeOOH catalytic sites for water dissociation, together with improved interfacial wettability. After 48 h of continuous operation, the transmembrane voltage of β-FeOOH-PDA@HNTs-BPM increased only from 0.94 to 0.98 V, indicating relatively stable electrochemical performance during the test period. Combined with the material characterization results, PDA-functionalized HNTs favor the dispersion of β-FeOOH and may promote its association with the support through multiple interfacial interactions. However, the specific contribution of these interactions to long-term stability requires further verification by post-operation structural and elemental analyses.

## Figures and Tables

**Table 1:** Membrane performance parameters.

Membrane	WU (%)	SD (%)	IEC (meq g^−1^)	Cond. (mS cm^−1^)	TN
Cation-exchange membrane	20–30	25–40	1.16	24.7	≥0.97
Anion-exchange membrane	30–40	30–50	2.10	31.0	≥0.98
FUJI-TYPE 12 AEM	30–40	30–50	1.1	–	–
FUJI-TYPE 12 CEM	20–30	25–40	1.0	–	–
Nafion 117	32–43	40–50	0.89	–	–

Note: WU, water uptake; SD, swelling degree; IEC, ion-exchange capacity; Cond., conductivity; TN, transport number. The symbol “–” indicates “not measured”.

**Table 2:** Transmembrane voltage at 50 mA cm^−2^ and I_lim1_ of the bipolar membranes.

Sample	Transmembrane Voltage at 50 mA cm^−2^ (V)	I_lim1_ (mA cm^−2^)
Blank-BPM	2.15	8.13
HNTs-BPM	2.04	7.98
PDA@HNTs-BPM	1.27	7.13
β-FeOOH-PDA@HNTs-BPM	0.94	5.80

**Table 3:** Fitting results of the electrochemical impedance spectra.

Sample	R_1_ (Ω)	R_w_ (Ω)	C (F)	λ (nm)	f_max_ (Hz)	τ_tr_ (μs)
Blank-BPM	0.627	3.873	2 × 10^−5^	31.9	4976	32
HNTs-BPM	0.650	2.950	2.13 × 10^−5^	29.9	6182	23.4
PDA@HNTs-BPM	0.680	2.290	3.23 × 10^−5^	19.7	7125	22.3
β-FeOOH-PDA@HNTs-BPM	0.780	0.980	6.49 × 10^−5^	9.82	12,110	13.1

## Data Availability

The raw data supporting the conclusions of this article will be made available by the authors on request.
